# Save for Safe: Effect of COVID-19 Pandemic on Consumers' Saving and Spending Behavior in China

**DOI:** 10.3389/fpsyg.2021.636859

**Published:** 2021-04-01

**Authors:** Xiaotong Jin, Yurou Zhao, Wei Song, Taiyang Zhao

**Affiliations:** ^1^Business School, Jilin University, Changchun, China; ^2^School of Philosophy and Sociology, Jilin University, Changchun, China

**Keywords:** COVID-19, saving and spending, risk perception, materialism, post-pandemic

## Abstract

In public health emergencies, people are more willing to save money rather than spending it, which is not conductive to economic development and recovery. Due to the absence of relevant research, the internal logic of this phenomenon is not clear. In the context of the COVID-19 pandemic, this study systematically explored whether and why public health emergencies stimulate consumers' preference for saving (vs. spending). We conducted two online surveys and used methods including stepwise regression analysis and bootstrapping to test the hypotheses. The first survey, with 1,511 participants from China in February 2020, indicates that the severity of emergencies has a significant positive impact on the populations' willingness to save (vs. spend). Risk perception plays a mediating role between the severity of emergencies and consumers' saving (vs. spending) willingness. Materialism plays a moderating role between risk perception and an individual's saving (vs. spending) willingness, individuals who are more materialistic have a lower saving (vs. spending) willingness when they perceive the risks of the pandemic. To verify the duration of the above effects, we conducted a follow-up survey consisted of 466 instances in August 2020. It is noteworthy that the above effects are not significant during the post-pandemic period. Thus, spending behavior in public health emergencies can be motived by reducing risk perception and increasing materialism. These findings can provide a valuable inspiration for public health, crisis management, and economic recovery during public health emergencies.

## Introduction

Emergencies can include natural disasters, accidental disasters, public health incidents, and social security incidents that occur suddenly, cause or may have the potential to cause serious harm to human life and environment and require emergency response measures. Since emergencies occur suddenly and have continuous impacts, they often cause serious damage to social order, life, public environment, and resources (Behar-Zusman et al., 2020; Wang and Su, 2020), and have a strong and far-reaching impact on the national economy (Muthuraman and Haziazi, 2020). The current COVID-19 pandemic has caused severe damage to the global economy. Therefore, economic recovery has become an important goal for governments globally. It is a known fact that economic recovery (during and after a pandemic) is inseparable from consumer consumption stimulation. However, contrary to this fact, people's consumption behaviors during pandemic usually tend to be disorderly and chaotic. One of the most typical behavior during the COVID-19 pandemic is panic buying (Arafat et al., 2020a), which results from individuals' fear of scarcity, losing control, and anxiety exacerbation (Arafat et al., 2020b; Yuen et al., 2020). In this case, individuals cope with threats by purchasing specific products, such as necessities (Jin et al., 2020; Sharma et al., 2020). However, in order to better deal with future uncertainties and risks, individuals' may also reserve resources and increase saving behavior. It is observed that individuals facing a pandemic save more than those who are not. For example, a survey in China showed that more than half of the country's households increased their savings and reduce their spending due to the pandemic; compared to the same period last year (when there was no pandemic) (Survey and Research Center for China Household Finance, 2020), the average savings of the younger generation increased by 34% (Fidelity International Alipay, 2020). Even Americans who are known for not loving saving money (De Rugy, 2004) show the same behavioral tendency. According to data from the Bureau of Economic Analysis (BEA) in April 2020 (Bureau of Economic Analysis, 2020), the personal savings rate in the United States hit an all-time high, reaching 33%, compared to the 12.7% that was observed in March 2020. While the savings rate is rising, the Visa report shows that consumer payments (of Americans) are plummeting (Cable News Network, 2020). These phenomena indicate the impact of COVID-19 pandemic on consumers' saving and spending behavior.

In fact, research in the field of economics has put forward the theory of uncertain consumption, which states that individuals will increase their savings and reduce spending under uncertain circumstances (Leland, 1968; Choi et al., 2001; Menegatti, 2001). However, this theory was put forward under ideal economic assumptions. Previous studies have also verified it at the macro level, failing to conduct research for a combination of specific situations and deep levels of individual psychology. Therefore, we raise the following questions: what is the psychological mechanism between the pandemic and an individual's saving and spending behavior? Is the effect long-term or short-term? Does everyone have the same behavior tendency? These issues are not yet clear and need to be resolved urgently. Our study is based on the background of the COVID-19 pandemic and introduces a risk perception theory to explore the above issues. Through the exploration of the above three issues, our study will provide suggestions on how to have a better understanding of consumer psychology and behavior during a pandemic. This analysis aims to provide a sound knowledge of consumerism during an emergency (like a pandemic) and to provide data that can help restore economy in such cases.

## Theoretical Framework

### Saving vs. Spending During the Pandemic

Saving and spending are not only the key nodes of individual consumption activities but are also important factors affecting economic development and growth. Therefore, saving and spending have been the focus of western economic research for a long time, and a series of classic theoretical frameworks have been formed based on them, such as the life-cycle hypothesis (Modigliani and Brumberg, 1954) and the precautionary savings hypothesis (Leland, 1968). Although these hypotheses provide theoretical support for economic model research, they are implemented at the macroeconomic level and fail to consider consumer psychology. Previous studies have found that consumers may increase preventive savings for dealing with impacts of unexpected emergencies (Drèze and Modigliani, 1972). According to a classical theory regarding uncertain conditions known as the “preventive saving hypothesis,” we can assume that the COVID-19 pandemic will increase the consumers' saving (vs. spending) behavior. This is because when consumers face uncertainty regarding the future, they make more precautionary savings (Kimball, 1990). At the same time, consumers expect the marginal utility of future consumption under uncertain circumstances to be greater than the marginal utility of future consumption under certain circumstances (Hau, 2002). Specifically, it can be expressed that the greater the uncertainty in the future, the greater the marginal utility of the consumers' expectation of future consumption. Therefore, they are more willing to increase precautionary savings to economically prepare for future consumption. Based on the above theory and logic analysis, the following hypothesis is proposed:

Hypothesis 1: The severity of the pandemic has a significant positive impact on the consumers' saving (vs. spending) behavior.

### Mediating Role of Risk Perception

Previous studies on the consumers' saving and spending behavior (under uncertain environments) were mainly explained from an economic perspective without verifying the psychological mechanism underlying such behaviors. We will explore the consumers' internal psychological mechanisms behind behaviors based on a risk perception theory. Risk perception refers to the psychological reaction of the public when, during an emergency, they perceive a threat to life, property, and other valuables (Slovic, 1987; Setbon et al., 2005). According to the psychometric paradigm, an individual's risk perception is closely associated with the severity of the disaster event (Burns and Slovic, 2012). As seen in a typical public health emergency, the influences of COVID-19 pandemic are large and widespread, posing a serious threat to life and property and the physical and mental health of residents (Saddique et al., 2020). Because they are facing a severe pandemic control situation, the residents believe that the external environment is full of uncertainty and may have serious consequences. Therefore, under the influence of the pandemic, the residents' risk perception will increase. Further, the social amplification framework of risk indicates that the social nature of emergencies will also expand or strengthen the residents' risk perception (Kasperson et al., 1988). Based on the above theory and logic analysis, the following hypothesis is proposed:

Hypothesis 2: The severity of the pandemic has a significant positive impact on an individual's risk perception.

According to the risk perception theory, when individuals perceive external risks, they perform various actions to reduce the risks (Shi and Kim, 2020). Studies have found that individuals may adopt conservative ways to deal with risks (Sandmo, 1970). For example, individuals may reduce risks by strengthening resource reserves and by strategically allocating their resources (Durante and Laran, 2016). Therefore, under the influence of the risk perception influenced by the pandemic, consumers may increase their willingness to save and reduce their willingness to consume, thereby increasing their control over the uncertain environment and ensuring that they can use their currency resources in the future as and when required. In addition, the pandemic triggers an individual's perception of death risk. Studies have found that when someone around an individual died, they are more inclined to give up short-term benefits for long-term benefits, thereby saving more money than spending it (Brown and Walensky, 2020). Based on the above theory and logic analyses, the following hypothesis is proposed:

Hypothesis 3: Risk perception has a significant positive impact on an individual's saving (vs. spending) behavior.

The severity of the COVID-19 pandemic can increase environmental uncertainty and thus, stimulate an individual's risk perception. The more severe the pandemic, the stronger the individual's risk perception. When risk perception is stimulated, people are usually more eager to adopt a conservative economic strategy (Eeckhoudt and Schlesinger, 2008; Menegatti, 2015); therefore, they may prefer saving money rather than spending it. Corresponding to this, the following hypothesis is proposed:

Hypothesis 4: Risk perception plays a mediating role between the severity of the pandemic and an individual's saving (vs. spending) behavior.

### Does the Effect Continue During the Post-pandemic Period?

Considering the effect of an individual's saving and spending behavior on economic recovery, studies on the effect of the severity of the COVID-19 pandemic on an individual's saving and spending behavior need to focus on the long-term effects, i.e., we need further studies to know if this effect still exists after the pandemic. By exploring an individual's psychology, we found that, in general, during a pandemic, consumers show an increase in their saving (vs. spending) behavior because they perceive risks. However, risk perception is a situational psychological variable (Slovic et al., 2004), when the situation changes, the individual's risk perception may change as well. According to the risk perception theory, the closer the disaster is, the higher the risk perception (Slovic, 2000). Thus, as the pandemic eases, the individual's risk perception is eliminated, and its impact on consumer saving and spending behavior also disappears. Therefore, the impact of the severity of a pandemic on consumers' saving and spending behavior is short-term and only exists during the pandemic. Based on the above theory and logic analysis, the following hypothesis is proposed:

Hypothesis 5: After the pandemic eases, the effects of a pandemic on an individual's saving (vs. spending) behavior will disappear.

### Moderating Role of Materialism

Although we have proved that the COVID-19 pandemic will increase an individual's perception of risk and boost their saving (vs. spending) behavior, in reality, there are still many people who deal with anxiety; these individuals ease their anxiety (caused by the pandemic) through consumption. Existing literature supports that fact the people respond to a pandemic by increasing their consumption. For example, Hill et al. (1997) believed that in the face of a death threat, individuals perceive the finiteness of life, thereby increasing consumption and reducing savings. Therefore, the following question arises: during a pandemic, who are more inclined to save and who are more inclined to consume? We find that materialism may be an important factor. Materialism refers to a value orientation of an individual, which expresses the extent to which the individual takes the acquisition of material ownership as an indication of achieved life goals. Materialistic individuals achieve success and happiness by pursuing material wealth and focus their entire lives on the pursuit and acquisition of property (Belk, 1985; Richins and Dawson, 1992; Chan and Prendergast, 2007). When individuals perceive the risk of the pandemic, individuals with high materialism always seek to own more things than others, as they believe goods can bring happiness. Therefore, they place more value on goods, and are more eager to acquire and retain properties (Ger and Belk, 1996). Compared to individuals who have a low materialistic approach, materialist individuals are more likely to obtain psychological comfort through the consumption of materials so as to relieve anxiety. This results in an increase in their spending behavior. In addition, materialism is not only an individual characteristic but also a cultural value generally accepted by society (Bauman, 1995; Kasser and Ryan, 1996). By supporting cultural values, materialist individuals can alleviate their mental insecurity in a risky environment (Pyszczynski et al., 1997), and therefore, their money-saving behavior will decrease. Based on the above theory and logic analysis, the following hypothesis is proposed:

Hypothesis 6: Materialism plays a moderating role between risk perception and an individual's saving (vs. spending) behavior. That is, compared with individuals with low materialism, individuals with high materialism show a decreased saving (vs. spending) behavior when they perceive the risk of a pandemic.

## Methods

### Participants and Procedures

We conducted two online questionnaire surveys on a professional platform named Credamo during and after the pandemic, which can provide large-scale data collection services and has been recognized by international top journals. The first survey was conducted during the COVID-19 outbreak (from February 10, 2020 to February 15, 2020). Credamo randomly distributed questionnaires in 31 provinces of China (excluding Hong Kong, Macao, and Taiwan) according to a quota of 50 copies in each province. A total of 1511 valid questionnaires were collected (with a recovery rate of 97.5%), covering 297 prefecture-level cities in 31 provincial-level administrative regions in China, which can accurately and comprehensively describe the psychology and behavior of Chinese citizens during the COVID-19 pandemic. The sample included 678 female participants (44.9%) and 833 male participants (55.1%), aged 18–79, and had a large share in income is below 3000 RMB per month (37.5%) and a significantly higher level education (67.5% of participants had completed a college or university program). To verify the duration of the effect, we conducted a follow-up survey after the pandemic eased. As the COVID-19 pandemic had eased in China as of August 2020, with almost no new cases, we randomly distributed a total of 500 follow-up questionnaires from August 3, 2020 to August 6, 2020 among participants who participated in the first survey and didn't change location, entered their information in the database, and recovered 466 questionnaires (with a recovery rate of 93.2%).

The questionnaire has passed the audit of Credamo, which guaranteed that it would not cause negative psychological effects on participants. As for the consent of participation, only those who agreed and volunteered to participate in surveys will access the questionnaire and corresponding remuneration. At the beginning of the questionnaire, we once again emphasized that “the survey results are only used for academic research, and the personal privacy of participants will be protected. If you agree and are participating voluntarily, start answering questions; if you disagree or are unsure, please exit.” Each participant received 10 RMB. This research was approved by the ethics committee of Jilin University. The ethics committee reviewed the study proposal and consent of the participants. Besides, to ensure the validity of the questionnaire, we set up test items to assess if participants completed the answers carefully. Questionnaires that failed this test were not included in our database. The linear interpolation method was applied to provide missing values. A CHERRIES-compliant reporting checklist is in Additional file.

The demographic information of the sample is shown in Table 1.

**Table 1:** Demographic information of sample (*N* = 1511).

**Items**	**Options**	**Sample**	**Percentage (%)**	**Items**	**Options**	**Sample**	**Percentage (%)**
Gender	Male	833	55.1	Age	<25	704	46.6
	Female	678	44.9		25–40	706	46.7
Income per month	<3000 RMB	566	37.5		>40	101	6.7
	3000–6000	543	35.9	Education	High school	351	23.2
	6000–9000	245	16.2		Bachelor's degree	1020	67.5
	>9000 RMB	157	10.4		Master's degree	140	9.3

### Measure

*Severity of pandemic* (SE). As the severity of pandemic varied in different Chinese cities, we considered using official pandemic indicators of different regions issued by the National Health Commission of the People's Republic of China. Specifically, in the first survey, we recorded the date on which the participants completed the questionnaire along with the names of the cities they lived in. Then, we searched the official pandemic indicators for the same timeframe and place and included them in the database to match the corresponding psychological and behavioral data. We chose four representative indicators: the cumulative number of confirmed cases per province (CNP), the number of new confirmed cases per province (NNP), the cumulative number of confirmed cases per city (CNC), and the number of new confirmed cases per city (NNC) to measure the SE and used them for hypothesis testing.

*Saving and spending willingness* (SA) was measured using a 7-point Likert scale, and we designed a three-item scale to measure the construct according to Durante and Laran (2016)'s study. The content of this scale was that “to what extent are you willing to use your money for savings (and not spending) at the moment,” “to what extent do you want to reduce spending money and increase savings in the future?” and “which one do you think is more important, saving or spending?”

*Risk perception* (RP) measurements were adapted from Katz et al. (2011), we aligned the measurements with a pandemic situation. The content of this scale was based on the following questions: “what do you think is the risk of COVID-19?” “how much do you think the COVID-19 pandemic poses a threat to health?” and “to what extent do you think COVID-19 has affected you?” All items used a 7-point Likert scale.

*Materialism* (MA) was measured based on Richins and Dawson (1992)'s study, which consists of 8 items. All items used a 5-point Likert scale ranging from 1 (very inconsistent) to 5 (very consistent).

*Control variables*. We also recorded the gender, age, education, and monthly income of the participants. As these variables may affect residents' saving and spending willingness, they were used as control variables.

## Results

### Reliability, Validity, and Measurement Model

We conducted a confirmatory factor and reliability analyses to test the reliability of the measurement model. The results are shown in Table 2. All the values of the constructs' Cronbach's α exceeded 0.700. Except for materialism [AVE (average variance extracted) = 0.462, nearly 0.500], the AVE of all other constructs exceeded 0.600, exhibiting sufficient reliability and convergent validity. We also tested the measurement model through confirmatory factor analysis. Considering the large sample size in this study, which might cause a chi-square expansion, we used the Bollen-Stine bootstrapping technique (5,000 bootstrapping samples) for correction (Bollen and Stine, 1992). The results showed that the model fitting indexes of the measurement model met the minimum requirements of χ2/df = 1.092, comparative fit index [(CFI) = 0.999], and root mean square error of approximation [RMSEA = 0.008] (See Table 2).

**Table 2:** Reliability and validity analysis (*N* = 1511).

**Variable name**	**Item**	**Standardize** **factor loading**	**C.R**.	**AVE**	**Cronbach's α**
SE	CNP	0.931	0.889	0.674	0.895
	NNP	0.964			
	CNC	0.659			
	NNC	0.681			
SA	SA1	0.766	0.818	0.602	0.805
	SA2	0.693			
	SA3	0.860			
RP	RP1	0.815	0.838	0.633	0.836
	RP2	0.842			
	RP3	0.726			
MA	MA1	0.731	0.872	0.462	0.871
	MA2	0.680			
	MA3	0.697			
	MA4	0.645			
	MA5	0.702			
	MA6	0.776			
	MA7	0.605			
	MA8	0.578			
Model Fit: χ2/df = 1.092, RMSEA = 0.008, CFI = 0.999, IFI = 0.999,TLI = 0.999					

*C.R., composite reliability; AVE, average variance extracted; RMSEA, root mean square error of approximation; CFI, comparative fit index; IFI, incremental fit index; and TLI, Tucker–Lewis Index*.

We used a diagonal matrix analysis method to test the discrimination validity of the variables. The intercorrelations of the variables and their AVE square roots are reported in Table 3. The off-diagonal numbers are the correlations. The AVE square roots are given in bold on the diagonal values. If the correlation coefficients between the variables are smaller than their AVE square root, it proves that the discrimination validity of variables is desirable. As shown in Table 3, the discrimination validity of the variables is good.

**Table 3:** Correlation and coefficient matrix (*N* = 1511).

	**SE**	**RP**	**MA**	**SA**
SE	**0.821**			
RP	0.036	**0.796**		
MA	0.147^**^	0.008	**0.680**	
SA	0.036	0.082^**^	−0.006	**0.776**

** *p < 0.01. The diagonal bold numbers are AVE square roots*.

### Hypothesis Tests

#### Analysis of Main Effect

We established a regression to verify whether the main effect is established and whether it is situational. The results showed that the SE had a significant positive impact on the SA during the pandemic (*β* = 0.051, *p* < 0.05) (see Table 4 for details); thus, the results verified H1. However, when the pandemic had eased, the SE had no significant influence on the SA (*β* = 0.008, *p* = 0.792) (see **Table 6** for details), indicating that the SE positively affected the SA only during the pandemic. Thus, H5 was verified.

**Table 4:** Regression test of the mediating effect during pandemic (*N* = 1511).

**Variable**	**SA**			**RP**	
	**Model 1**	**Model 2**	**Model 3**	**Model 4**	**Model 5**
Gender (female =0)	−0.060^*^	−0.063^*^	−0.065^*^	0.028	0.025
Age	0.109^***^	0.106^***^	0.104^***^	0.040	0.037
Education	0.074^**^	0.074^**^	0.072^**^	0.038	0.039
Monthly income	−0.111^***^	−0.119^***^	−0.110^***^	−0.115^***^	−0.124^***^
SE		0.051^*^	0.047		0.053^*^
RP			0.070^**^		
*R^2^*	0.026	0.029	0.034	0.011	0.014
*Adj.R^2^*	0.024	0.026	0.030	0.008	0.010
*F*	10.187^***^	8.936^***^	8.733^***^	4.198^**^	4.178^***^

* p < 0.05,

** p < 0.01, and

*** *p < 0.001*.

#### Analysis of Mediating Effect

We applied the regression analysis method proposed by Baron and Kenny (1986) to test the mediating role of RP between the SE and the SA during the pandemic. The results are shown in Table 4. The SE had a significant positive impact on the RP (*β* = 0.053, *p* < 0.05) and the RP had a significant positive impact on the SA (*β* = 0.070, *p* < 0.01). H2 and H3 were verified. After the addition of RP, the SE had no significant impact on the SA (*β* = 0.047, *p* > 0.05), indicating that RP played a fully mediating role between the SE and the SA during the pandemic. Thus, H4 was verified.

We used a bootstrap procedure to re-verify the mediating effect during the pandemic (Preacher et al., 2007). This procedure computed a 95% confidence interval (CI) for the indirect and direct effects through 5,000 sampling. If a CI does not include 0, it indicates that the effect is significant. The results are shown in Table 5. The CI of the total effect [*β* = 0.082, 95% CI: (0.001, 0.163)] and the indirect effect [*β* = 0.006, 95% CI: (0.001, 0.016)] did not include 0, while the CI of the direct effect [*β* = 0.076, 95% CI: (−0.006, 0.157)] included 0, indicating that RP played a significant mediating role between the SE and the SA during the pandemic. Thus, H4 was verified.

**Table 5:** Results of mediating effect during pandemic (*N* = 1511).

**Path**	**Effect**	**Standardized estimate**	**S.E**.	**95% Confidence intervals**	
				**LICI**	**ULCI**
SE->SA	Total effect	0.082	0.042	0.001	0.163
SE->RP->SA	Indirect effect	0.006	0.004	0.001	0.016
	Direct effect	0.076	0.042	−0.006	0.157

We used the same method to test the mediating role of RP between the SE and the SA during the post-pandemic period. The results are shown in Table 6. We observed that the SE had no significant positive impact on RP (*β* = 0.013, *p* > 0.05). The RP still had a significant positive impact on the SA (*β* = 0.124, *p* < 0.01), indicating that after the pandemic eases, the effect of SE on SA would disappear; this is because an individual's risk perception ceases to exist after the pandemic. Thus, H5 was verified again.

**Table 6:** Regression test of mediating effect after pandemic (*N* = 466).

**Variable**	**SA**			**RP**	
	**Model 1**	**Model 2**	**Model 3**	**Model 4**	**Model 5**
Gender (female = 0)	−0.089	−0.089	−0.088	−0.008	−0.009
Age	−0.131^**^	−0.130^**^	−0.136^**^	0.047	0.048
Education	−0.052	−0.052	−0.051	−0.007	−0.007
Monthly income	−0.052	−0.055	−0.056	0.009	0.004
SE		0.013	0.010		0.022
RP			0.124^**^		
*R^2^*	0.034	0.034	0.049	0.003	0.003
*Adj.R^2^*	0.026	0.024	0.037	−0.006	−0.008
*F*	4.052^**^	3.250^**^	3.974^***^	0.293	0.274

* p < 0.05,

** p < 0.01, and

*** *p < 0.001*.

#### Analysis of Moderating Effect

This study uses hierarchical regression to analyze moderating effects. If the interaction item is significant, it means that the moderating effect is supported. Before the final calculation, all data were centralized to reduce the possible multicollinearity of the data. In the relationship between the SE and the RP, the M3 result showed that the value of RP × MA was −0.062, the *T*-value was −2.432, *p* < 0.05 (*R*^2^ = 0.035, Adjust *R*^2^ = 0.031, Δ*R*^2^ = 0.004, F-change = 5.914), indicating that MA plays a significant negative moderating role between RP and SA. Thus, H6 was verified (see Table 7).

**Table 7:** Regression test of moderating effect during pandemic (*N* = 1511).

**Variable name**	**SA**					
	**M1**		**M2**		**M3**	
	**β**	**T**	**β**	**T**	**β**	**T**
Sex	−0.060^*^	−2.284	−0.062^*^	−2.366	−0.060^*^	−2.283
Age	0.109^***^	3.999	0.106^***^	3.873	0.104^***^	3.800
Monthly income	−0.111^***^	−3.931	−0.102^***^	−3.619	−0.101^***^	−3.567
Education	0.074^**^	2.808	0.071^**^	2.708	0.070^**^	2.666
RP			0.072^**^	2.839	0.074^**^	2.886
MA			−0.002	−0.085	−0.003	−0.126
RP×MA					−0.062^*^	−2.432
*R*^2^	0.026		0.032		0.035	
Adjust *R*^2^	0.024		0.028		0.031	
*F*	10.187^***^		4.032^*^		5.914^*^	

* p < 0.05,

** p < 0.01, and

*** *p < 0.001*.

In order to further test the results of the regression analysis, we used a bootstrap method (sampling times of 5,000, 95% confidence interval, model 14) for analysis. Only when the value of materialism was equal to the average [*β* = 0.006, 95% CI: (0.001, 0.017)] and one standard deviation was lower than average [*β* = 0.011, 95% CI: (0.002, 0.028)] was the indirect role of the SE on the SA significant. Thus, H6 is verified (see Table 8).

**Table 8:** Result of moderating effect during pandemic (*N* = 1511).

**Moderating construct**	**Path**	**Value**	**Effect**	**95% Confidence intervals**	
				**LICI**	**ULCI**
MA	SE->RP->SA	2.304	0.011	0.002	0.028
		3.045	0.006	0.001	0.017
		3.786	0.001	−0.005	0.011

## General Discussion

This study explored the impact of the severity of the COVID-19 pandemic on an individual's saving and spending behavior and its psychological mechanism. The results of the questionnaire survey are as follows: (1) the severity of pandemic is positively associated with an individual's saving (vs. spending) behavior; (2) the perceived risk plays a fully mediating role in the impact of the severity of pandemic on the saving and spending behavior i.e., the more severe the pandemic, the greater the risk perception of individuals and the higher their willingness to save (vs. spend); (3) after the pandemic eases, the effect of pandemic severity on individual's saving (vs. spending) behavior will disappear; (4) materialism plays a moderating role between the risk perception and the individual's saving and spending behavior. Compared to individuals with low materialism, individuals with high materialism have a lower saving (vs. spending) willingness when they perceive the risks of a pandemic. Therefore, this study provides sufficient empirical support for the relationship between the pandemic and the consumers' saving and spending behavior.

### Theoretical Contribution

By exploring the relationship between the pandemic and an individual's saving and spending behavior, this research provides references for the study of emergencies and emergency management. An important innovation of research is the simultaneous analysis of the short-term and long-term effects of a pandemic on an individual's saving and spending behavior. Previous studies on the impact of emergencies mostly used secondhand data to verify this fact, which could not directly reflect the current psychology and psychological changes of residents; thus, previous studies could not verify the continuity in peoples' behaviors during emergencies. Through investigations during and after the pandemic, we verified the sustainability of the effects of the pandemic on an individual's saving and spending behavior, which not only enriches and completes the relevant theories but also provides methodological references for related research.

Our study also made theoretical contributions to research in the fields of economics, psychology, and public health. First, the existing research on saving and spending under uncertain conditions only remains at the level of economic theory and fails to penetrate the consumers' psychology. We place saving and spending behavior in the context of a public health emergency and explain the mechanism between the pandemic and the consumers' saving and spending behavior from the dual perspectives of external environment and internal psychology. This helps us to deepen the exploration of relevant economic theory.

In the field of consumer psychology and behavior, research on saving and spending has gradually become a hot topic. However, related research is still in the initial stage and needs further exploration. We explored the impact of the pandemic on the saving and spending behavior of consumers' and found that risk perception plays a mediating role in this behavior. Additionally, materialism plays a moderating role in determining the behavior, which can be seen as a supplement to related content. At the same time, although materialism has become a universal cultural value, the term materialism still has a derogatory meaning, and research is mostly based on its negative influences. We discover the positive side of materialism i.e., materialism can provide individuals with psychological support in an unsafe and risky environment. The conclusion provides empirical support for the consumer behavior during emergencies and for the development of related theories about risk perception and materialism.

In the field of public health, previous studies of public health have focused primarily on infection prediction and health behaviors, however in public health emergencies, panic behavior need more attention. Recent studies have found that the pandemic cause more panic buying, for example, individuals may increase their purchases of necessities. Our study suggests that the savings behavior under COVID-19 pandemic can be regarded as a panic response behavior of individuals for future risks, which provides a new perspective for public health research. Also, most of the risk perception of emergencies has been verified under catastrophic emergencies. Our results once again verify the application of risk perception theory in public health emergencies, which can help grasp the public's psychology during the pandemic and contribute to build a reliable research framework for risk perception theory.

### Practical Implications

Based on the COVID-19 pandemic, we analyze the impact of public health emergencies on an individual's saving and spending behavior in China through theoretical and empirical analysis. These research results have an important reference significance for the government's crisis response and emergency management. On the one hand, the findings verified that the materialism can moderate the approach which people cope with public health emergency, thereby promote individuals' normal consumption behavior, alleviate their panic behavior. On the other hand, we found that consumers, during the pandemic, are more inclined to save rather than consume. Although the effect is not long-term, from a socioeconomic perspective, this approach is not conducive to the recovery and development of the economic market. Therefore, the government can also take corresponding measures to promote their resident's consumption. Chinese government have taken corresponding measures to subsidize people's lives and stimulate consumption, such as issuing consumption coupons. This study found that individuals' risk perception of the pandemic is the key factor to affect their propensity to save and consume. Therefore, the government can popularize scientific knowledge of the pandemic to enhance people's understanding of the circumstance to comfort the residents, reduce their risk perception, and promote consumption. In general, the Chinese government should build confidence, firmly believe that the impact of the pandemic is short-term, strive to restore normal economic and social order, and promote stable and healthy economic development.

### Research Limitations and Future Research Directions

This study has some limitations. First, we conducted research in China, which may be affected by social-cultural factors and show certain particularities. In the future, research can focus on the comparison of different countries with different social cultures. Second, this study selected the subjects randomly. In fact, the key individual of the family, who have more power to decide on saving money or spend it, need to be considered. Future study can pay attention to this and conduct in-depth research.

## Data Availability Statement

The original contributions presented in the study are included in the article/Supplementary Material, further inquiries can be directed to the corresponding author.

## Ethics Statement

The studies involving human participants were reviewed and approved by Ethics committee of Jilin University. The patients/participants provided their written informed consent to participate in this study.

## Author Contributions

XJ and YZ devised the project, the main conceptual ideas, and proof outline. XJ and TZ collected the research data and revised the manuscript. YZ and WS analyzed the sequencing data and wrote the initial manuscript. All authors contributed to the article and approved the submitted version.

## Conflict of Interest

The authors declare that the research was conducted in the absence of any commercial or financial relationships that could be construed as a potential conflict of interest.
