# Supplementary material for: Save for Safe: Effect of COVID-19 Pandemic on Consumers' Saving and Spending Behavior in China
Source: Front Psychol. 2021 Apr 1;12:636859. doi: 10.3389/fpsyg.2021.636859 (PMC8047313; doi:10.3389/fpsyg.2021.636859)
Supplement: Supplementary file 1 [file Table_1.DOCX]

**CHERRIES-Compliant Reporting Checklist**

| Item Category | Checklist Item | Response |
| --- | --- | --- |
| Design | describe survey design | We designed this survey for a random sample of Chinese residents from 31 provinces. |
| IRB approval and informed consent process | IRB approval | The Institutional Review Board at Jilin University reviewed this study. |
|  | informed consent | We placed informed consent language approved by the IRB at the beginning of the survey, only those who agreed and volunteered to participate in surveys will access the questionnaire and corresponding remuneration. At the beginning of the questionnaire, we once again emphasized that “the survey results are only used for academic research, and the personal privacy of participants will be protected. If you agree and are participating voluntarily, start answering questions; if you disagree or are unsure, please exit.” |
|  | data protection | We would not disclose personal information. |
| development and pre-testing | development and testing | The survey was developed based on mature scales. In most studies, the reliability and validity of scales have been tested to be good， therefore, we didn’t pilot tested the survey. |
| recruitment process and description of the sample having access to the questionnaire | open survey versus closed survey | This survey was an open survey. |
|  | contact mode | The initial contact with the potential participants was made through Credamo platform, which can provide large-scale data collection services and has been recognized by international top journals. |
|  | advertising the survey | We conducted two online questionnaire surveys during and after the pandemic. In the first survey, Credamo randomly distributed questionnaires in 31 provinces of China (excluding Hong Kong, Macao, and Taiwan) according to a quota of 50 copies in each province. In the second survey, considering that participants who moved location may be affected again by the pandemic severity in a new location, we used Credamo to randomly distribute the questionnaire to 500 participants who participated in the first survey, whose location had not changed between February and August. |
| survey administration | web/email | This survey was a web survey hosted on Credamo. |
|  | context | The survey covered 297 prefecture-level cities in 31 provincial-level administrative regions in China, which can accurately and comprehensively describe the psychology and behavior of Chinese citizens during the COVID-19 pandemic. |
|  | mandatory/voluntary | This survey was voluntary. |
|  | incentives | We offered each participants 10 RMB for incentives. |
|  | time/date | Data were collected during February 10, 2020 to February 15, 2020 and August 3, 2020 to August 6, 2020. |
|  | randomization of items or questionnaires | This survey had no randomization or alternation. |
|  | adaptive questioning | There is no adaptive questioning in the survey. |
|  | number of items | This survey included 20 items. |
|  | number of screens (pages) | This survey included 4 screens. |
|  | completeness check | This survey did not include any completeness check. |
|  | review step | This survey did not present participants with a summary of the responses. However, participants could use a "previous page" button to return to earlier pages and select different answers. |
| response rates | unique site visitor | The survey platform Credamo record respondents' IP addresses, and we determine unique visitors to prevent repeated answers. |
|  | view rate | The view rate cannot be calculated without a unique site visitor number. |
|  | participation rate | The participation rate cannot be calculated without a unique site visitor number. |
|  | completion rate | In the first survey, the completion rate was 97.5%. In the first survey, the completion rate was 93.2%. |
| preventing multiple entries from the same individual | cookies used | We used cookies to assign unique user identifiers. |
|  | IP check | We used participants' IP addresses to limit the number of entries. |
|  | log file analysis | We investigated log files，and exclude duplicate responses, based on log file analysis. |
|  | registration | This survey was open, so this item does not apply. |
| analysis | handling of incomplete questionnaires | We analyzed partially completed questionnaires. |
|  | questionnaires submitted with an atypical timestamp | We did not impose a cut-off time. |
|  | statistical correction | We did not adjust for the non-representative sample. |
